# Critical Literature Review on Clinical Presentation of Oncocytic Thyroid Carcinoma with Immunoendocrine Complications and Unpredictable Outcome: Myths, Facts, and Their Overinterpretation

**DOI:** 10.3390/biomedicines14061335

**Published:** 2026-06-12

**Authors:** Przemyslaw Zdziarski

**Affiliations:** 1 Department of Pharmacology, Faculty of Medicine, Wroclaw Medical University, 50-345 Wroclaw, Poland; zdziarski@oil.org.pl; 2 PRION Private Institute of Nature, ul. Jana Mikulicza-Radeckiego 2, 50-385 Wroclaw, Poland

**Keywords:** oncocytic/Hürthle cell carcinoma/adenoma, symptoms, diagnosis/diagnostic chain, bias, standardization, neuroendocrine cancer, paraneoplastic syndrome, reverse flip-flop phenomenon, hypercalcemia

## Abstract

**Objectives**: Endocrine neoplasms, as a general rule, show systemic, neuro-inflammatory and metabolic consequences, known as paraneoplastic syndrome. The comorbidity of thyroid tumors with neurological and autoimmune diseases prompt looking for common neuro-immuno-endocrine mechanisms of these disorders. While most TCs are well described, there is a gap in the literature after the isolation of oncocytic/Hürthle cell carcinoma (HCC), as a unique type due to immunoendocrine and metabolic features (low TSH-receptor expression and radioiodine avidity). The aim of this study was to collect clearly defined reports of HCC (as a separate entity) and to attempt determining common clinical symptoms and the usefulness of various diagnostic techniques (comprehensive critical review). This may be an introduction to modern treatment (patient-centered care) since the main cause of mortality is not local progression or metastases. **Results**: Until now, due to misnomenclature and data misinterpretation, HCC has been treated according to general standards (with overuse of TSH-ST and RIA). High thyroglobulin level, decreased total thyroxin (with normal FT3 and spontaneous decrease in TSH), hypercalcemia, as well as the “reverse flip-flop” phenomenon, as common symptoms, indicate the neuroendocrine origin of HCC. Sparse, well-documented lymph node metastases are another feature, although from few studies. Most studies omit the N stage. Whole-body ^131^iodine and ^18^F-fluorodeoxyglucose scintigraphy may be useful before FNAB. Fine-needle aspiration biopsy (FNAB), as a “gold standard” in early diagnosis of thyroid nodules, delays HCC diagnosis because of the inability to determine a benign/malignant nature. **Conclusions**: Final HCC outcome may be affected by various overlapping immunoendocrine factors (paraneoplastic effects). Due to very few thyroid function tests performed in HCC, we have proposed a set of basic laboratory analyses, core biopsy in HCC differentiation, and diagnostic chain for standardization. According to the review, adaptation and treatment of HCC based on existing standards for other thyroid cancers seem to be insufficient, and the risks outweigh the benefits. The key recommendations resulting from the 5th edition of the WHO Classification of Endocrine Neoplasms are only the beginning of refuting many myths and biases.

## 1. Introduction

Historically, Hürthle cell adenoma (HCA) and carcinoma (HCC) were regarded as variants of follicular adenoma and thyroid carcinoma (FA and FTC), respectively. HCC/HCA was separated as an independent class relatively recently [1,2]. Hürthle cell adenomas and carcinomas were nominated as a distinct entity in the 2017 WHO (World Health Organization) classification, since they have biological behaviors and molecular profiles that differ from those of their counterparts among follicular tumors, currently named as oncocytic adenoma (OA) and oncocytic carcinoma (OC) in 5th edition of the WHO Classification of Endocrine Neoplasms [3,4].

HCC has a different genetic profile from other types of thyroid cancer (TC). Therefore, in 2017, the International Agency for Research on Cancer (IARC) described Hürthle cell (oncocytic) tumors in a new chapter of the 4th edition of the WHO Classification of Tumors of Endocrine Organs, acknowledging their peculiar biological and clinical features [3]. The new classification included [3,4]: (1) an oncocytic variant of PTC (ICD-0 morphology code 8342/3) and (2) Hürthle (oncocytic) cell tumors, that is (a) Hürthle cell adenoma (HCA) (8290/0) and (b) Hürthle cell carcinoma (HCC) (8290/3).

In the 5th edition of the WHO Classification of Thyroid Tumors, Hürthle cells are definitively referred to as oncocytes. All oncocytic tumors have an arbitrarily set minimum proportion of oncocytes at 75% [4]. The reclassification is mainly at the molecular/biological level [5], but HCC is a rare and understudied cancer with unknown prognosis. Despite advances in molecular characterization, usually based on reanalysis of historical samples (sometimes non confirmed) [6], the same approach cannot be applied to clinical observations (i.e., symptoms). Demographic data such as age and gender are insufficient and mainly show simple correlations, not a pathway.

To date, despite a continuously growing interest in TC and a large number of scientific reports regarding HCC, the literature and clinical presentation remain limited. This paper is the first attempt of a critical review of available data found in the literature.

Clearly, the main and highest priority was collecting unquestionable facts from various publications and their critical review (not looking for statistical relationship).

## 2. Literature Search Strategy

This paper is a review of texts published within the last 10 years (2015–2025; the latest literature update: January 2026) obtained in electronic form from the MEDLINE/PubMed database and Cochrane Library. While browsing the papers to be analyzed, the following keywords were used: “oxyphil cells” OR (“oxyphil”) AND (“cells”) OR “oxyphil cells” OR (“Hurthle” AND “cell”) OR “Hurthle cell” OR “Hürthle Cell” “oncocytic thyroid cancer,” “oncocytic adenoma” (OA) and “oncocytic carcinoma” (OC), “Hurthle Cell Cancer” or “Hurthle Cell Carcinoma,” and “oncocytic head and neck cancer” in studies published between 2015 and 2025.

Initially, 652 results were found. Due to difficult spelling, many works contained a misnomer: Hurtle instead of Hurthle/Hürthle (for example, ref. [7]). Despite this formal deficiency, these works (76 in total) were included in the analysis as they often contained important information.

**Figure 1:**
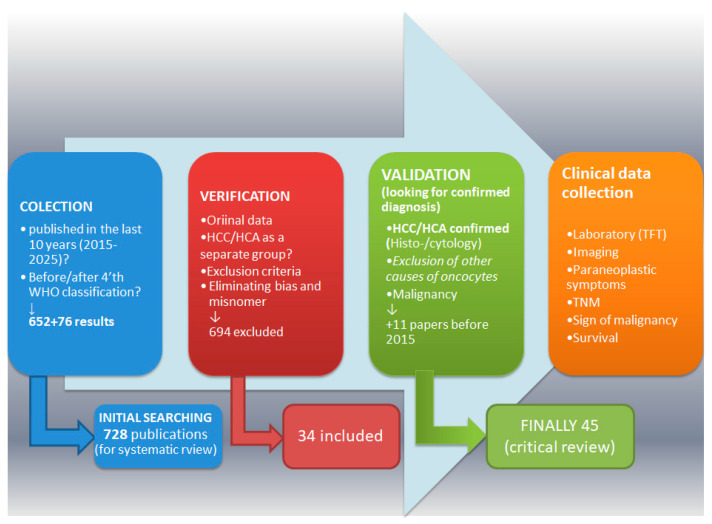
Search and selection strategy for collection of clinical data. After initially performing a systematic review of papers published between 2015 and 2025, 652 publications were selected. Additionally, after adding the “Hurtle cell” misnomer, 76 publications were included in the analysis as they often contained important information. After the verification of 728 publications (elimination of bias, review papers, or meta-analyses), most of them were excluded (694). Therefore, the time criterion, in particular related to the 4th edition of the WHO classification, was withdrawn. There were well-documented (unquestionable reports) of thyroid cancer from Hürthle cells. Some clinical data were obtained from 11 papers after consecutive validation (i.e., paper analysis and unquestionable confirmation of diagnosis). Finally, patient data were obtained from the period of 1946–2026. Some studies raising methodological doubts and inconsistent nomenclature are cited as examples of bias and a source of numerous inconsistencies (see graphical abstract). **Abbreviations**: HCA/HCC—Hürthle cell adenoma/carcinoma; TFTs—thyroid function tests; TNM—TNM classification of malignant tumors.

Conference materials and unpublished PhD theses were not included. Finally, full texts were analyzed, and methodological errors constituted an additional exclusion criterion at this stage (see Section *Data Analysis (Bias Elimination): An Attempt to Collect and Unify Good Clinical Practices*). Furthermore, analysis of references helped to include 3 more papers. The selection process is presented in Figure 1. Although the 5th edition of the WHO Classification of Endocrine Neoplasms introduced the preferred terms oncocytic adenoma (OA) or carcinoma (OC) instead of Hürthle cell adenoma or carcinoma, most of the reviewed papers come from an earlier period and contain this synonymous keyword (used in this paper).

### Data Analysis (Bias Elimination): An Attempt to Collect and Unify Good Clinical Practices

Initially, based on the title and a preliminary analysis of the abstract, it was investigated whether the selected texts concerned the subject of the review. The results were unexpected and raised concerns about validity. For example, some publications misused Hutrle instead of Hurthle cell cancer. Due to numerous inaccuracies, primarily including the titles of the manuscripts (which significantly differed from the data in the abstracts), we introduced exclusion criteria for papers that: (i) concerned species other than human, (ii) were available in languages other than Polish, Russian, German and English, (iii) had substantial methodological errors in the diagnosis, and (iv) uses non-precise nomenclature (e.g., misnomer).

Publications in which the keywords were mentioned only in the introduction or discussion and did not constitute a separate HCC/HCA category described in the results were considered useless. The vast majority of these were works from before 2013, the period when oncocytic tumors began to be identified at the molecular level [1,2].

Review papers, meta-analyses, and statistical studies in which the reliability of the data was not strictly checked were used as examples of bias. Even publications with “Hürthle cell carcinoma” in the title provided a number of comparisons and descriptions that actually concerned FTC or DTC [8], basic demographic characteristics [9], or cytological features [10] without clinical data.

The seven key problems in the comparability and repeatability of most publications were: (1) retrospective nature, and therefore very selective assessed parameters (e.g., TSH, only T stage); (2) significant time difference between the date of research and publication (even decades) [6]; (3) using anthropometric data (e.g., gender, age, race, BMI) without reference to direct factors (especially hormone levels, mitochondrial disease), i.e., statistical relationship or contrary to pathway; (4) building statistics based on medical history and qualitative data (e.g., presence of diabetes, exposure to radiation) [9]; (5) ambiguous impact of previous interventions on the presented results (e.g., vitamin D, T4 supplementation, surgery) [10,11]; (6) heterogeneity of histopathological data, i.e., cytological diagnoses are not the same level as postoperative ones; and (7) no distinction was made between oncocytic adenoma (OA) and oncocytic carcinoma (OC), (i.e., HCC and HCA), with unclear criteria for malignancy.

Regardless of the terminology, these limitations were so significant that they eliminated most of the publications. Therefore, the initial intention—a systematic review of 728 papers—would be biased, and the work became a critical review of unquestionable data from 45 original reports. The time criterion was abandoned because the retrospective reports, although published recently (2003), were based on earlier data (e.g., 1946–2003) [6]. The aim of our work was not to create another meta-analysis but to critically review the literature on HCC as a separate and precisely described disease entity. In the thicket of information, it is very difficult to reject myths and half-truths. However, it shows how little we know about a given topic.

## 3. Oncocytic Thyroid Cancer Diagnosis—Standardization as the Highest Scientific and Clinical Need: Precision Medicine

Approximately 200 million people have been diagnosed with thyroid disease worldwide [11]. Several biases, dogmas, and paradigms are described in the diagnosis of TC. Clinical, political, and patient demands for simple, cost-effective, and successful therapies have prompted preemptive intervention, highly specialized teams (narrow oncological units), and limited to a small area approaches rather than holistic, comprehensive medicine.

Although the first step and basic element in the diagnosis of TC is a physical examination, a small percentage of patients (usually with clear goiter and lymphadenopathy) are referred to specialists (e.g., endocrinologists or oncologists). Therefore, the initial selection is based on ultrasonography (US), with biochemical and hormonal tests performed by a primary care doctor, but also on other considerations that are of great statistical significance in public health (e.g., “women’s health checkup”, latest guidance—see below).

### 3.1. Laboratory Data

**Figure 2:**
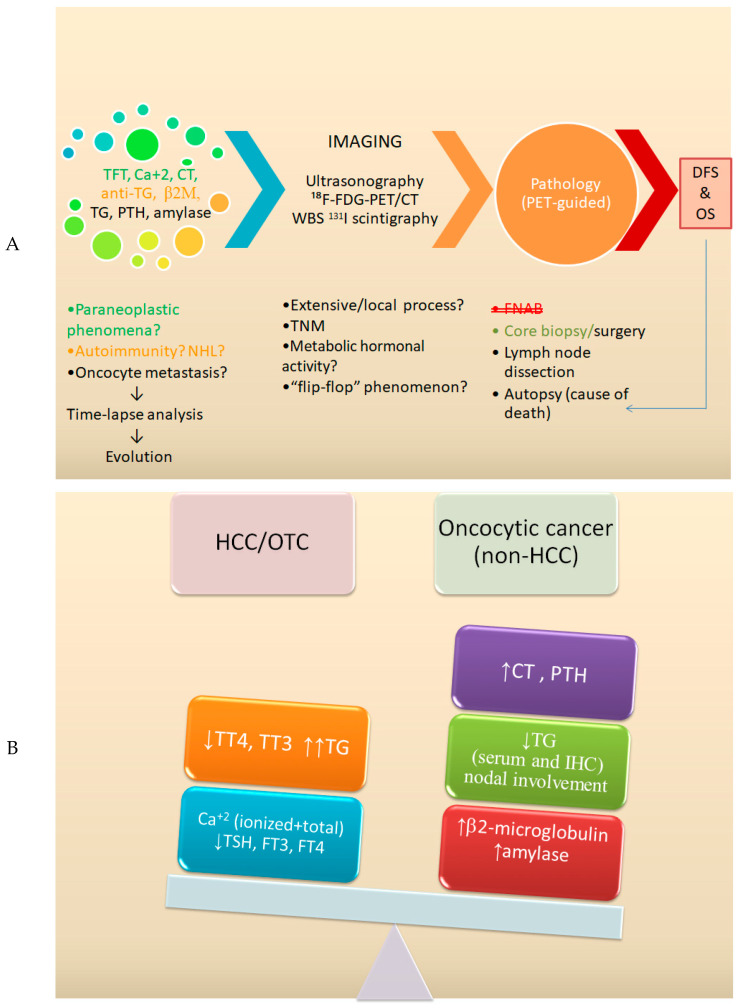
Precision medicine and proposed diagnostic chain in: initial step (**A**) and differentiation of Hürthle cell (oncocytic) carcinoma (**B**). Precision medicine requires an evaluation of overall homeostasis, including hormonal balance (i.e., thyroid function tests—TFTs) and autoimmunity (i.e., anti-TG). If HCC is suspected based on the clinical picture and paraneoplastic symptoms, further diagnostic imaging-guided observation, techniques, and histopathology. In the new perspective, diagnostic-chain whole-body radioiodine scans (WBS ^131^I) and PET (i.e., [^18^F]FDG uptake) precede biopsy with various techniques (surgery, fine-needle aspiration (FNAB), or core biopsy). Contrary to previous clinical practice in TC, if HCC is suspected, a core biopsy (not FNAB) is recommended. **Abbreviations:** Anti-thyroglobulin antibody (anti-TG); β2-microglobulin (β2M); calcitonin (CT); disease-free survival (DFS); overall survival (OS); fine-needle aspiration biopsy(FNAB); Hürthle cell carcinoma (HCC); immunohistochemistry (IHC), non-Hodgkin lymphoma (NHL); oncocytic thyroid Cancer (OTC); parathyroid hormone (PTH); ^18^F-fluorodeoxyglucose positron emission tomography/computed tomography (FDG-PET/CT); thyroid function tests (TFTs); thyroglobulin (TG); TNM classification of malignant tumors (TNM); total and free tetra-/tri-iodothyronine (TT4/3 and FT4/3); whole-body ^131^iodine scintigraphy (WBS).

Laboratory tests are important in searching for symptoms and homeostasis typical for TC, especially HCC. Several test results are quite important in clinical practice but they are underutilized. Due to the need for standardization and validation in TC, thyroid function tests (TFTs) should be initially used (Figure 2A), as well as for HCC differentiation (Figure 2B): (1) human thyroglobulin (TG) (serum and in immunochemistry (IHC); (2) total levels of thyroxine and triiodothyronine (TT4 and TT3, respectively), which are an indicator of direct production (unlike FT3 and FT4, which correlate with the functional thyroid state); (3) calcitonin (CT), together with total/ionized calcium and parathyroid hormone (PTH) level (for differentiation with medullary and parathyroid cancer); (4) thyroid-stimulating hormone (TSH) (of course together with TT3 and TT4); (5) β2-microglobulin and serum amylase levels (for differentiation with thyroid lymphoma and Warthin’s tumor); and (6) glucose and electrolytes (time-lapse analysis) (in paraneoplastic phenomena).

According to the latest guidance, if thyroid dysfunction is suspected, it is recommended to measure thyroid-stimulating hormone (TSH) alone in adults and then free thyroxine (FT4) only if TSH is above the reference range or together with free tri-iodothyronine (FT3) if TSH is below normal [12]. This is crucial in the case of observed increased mortality associated with thyroid hormone overuse (see Section 5.2. *Thyroid-Stimulating Hormone Suppression Therapy (TSH-ST) Overuse*) [13]. 

Unfortunately, most publications, including medical records, contained quite limited and unique data, making comparisons to other publications difficult. This is a significant limitation, especially in the era of common meta-analyses, the scientific value of which is primarily the result of uniform, repeatable results obtained using a similar methodology in individual publications [14]. Following the latest guidance, in general practice, mainly TSH tests are performed, sometimes enriched with FT4, and much less frequently with FT3 [12]. Noteworthy, these TFTs are not a direct echo of thyroid hormone production but instead reflect the biological effects of hormones (free hormone correlates with the functional thyroid state). The final result of FT3/FT4 is influenced by carrier proteins or the displacement of T3/T4 from protein (e.g., TBG, albumin). Furthermore, even HCC case reports are based on time-lapse analysis of TSH and FT4, then, after detecting any deviations (tests that prompted urgent referral to endocrinologists), the diagnosis is supplemented with FT3 and other hormones. Of note, in HCC, the decrease in FT4 is 3-fold and that in total thyroxin is 6-fold compared to normal [15]. Even in developed countries, patients after HCC surgery (partial thyroidectomy) were monitored in a limited way over the years [15]. Sometimes a patient with no initial assessment of FT3/FT4 (nor T3/T4) after partial thyroidectomy received radioiodine therapy or TSH-ST (thyroid-stimulating hormone suppression therapy up to 200 μg/day), with serious complications and adverse drug reactions (ADRS), e.g., T3 thyrotoxicosis [16]. This explains why we know little about the symptoms of HCC itself (summarized in Table 1), and the data are conflicting (usually from patients after many unmonitored interventions).

The non-standardized diagnostic chain appears to be the number one cause of discrepancies in the literature. The clinical routine is based on abnormalities in the ultrasound image (only the thyroid gland, excluding lymph nodes, salivary glands, and distant organs, e.g., the kidney), then FNAB, and in case of uncertainty, surgery. Therefore, most studies pay no attention to basic markers (e.g., testing glycemia or calcium levels), endocrine homeostasis (e.g., PTH, CT, TT3/4), or immunity (TG together with anti-TG), especially standard oncological TNM staging [17].

**Table 1:** Clinical and laboratory data (symptoms) of patients with Hürthle cell carcinoma: the need for standardization and validation of thyroid function tests and the diagnostic chain.

Symptom	Comments *	Reference
Hypercalcemia **	Without bone involvement (necessary PTH, CT, AP assessment)	[15] [16] [18]
Normal FT3 (free tri-iodothyronine) (various units and reference range)	5.9 [3.8–6.0 pmol/L] * 3.05 pg/mL [2.6–4.8]	[15] [19]
Free and **total** thyroxine (**FT4** and **TT4**) (various methods, units, and reference ranges)	Low i.e., 2.7 and **12.3 ** [**FT4** 9–20 pmol/L; **TT4** 69–141 nmol/L] or Normal **FT4** 1.05 [0.8–2.7 ng/mL]	[15] [19]
Thyroid-stimulating hormone (TSH) (non-standardized reference range)	Progressively low 1.82⟶0.59 mIU/L [0.35–3.5] or Normal 1.52 mIU/L [0.4–4.2]	[15] [19]
High level of thyroglobulin (TG) [0–3 ng/mL]	Median 163 [ng/mL] (31 HCA patients) 638.5 (27 HCC patients) vs. 2895 (FTC patients) 60% of patients (22/37) (TG range = 80 ± 25 ng/mL) >38,000 ng/mL [<55] TG (−) oncocyte metastasis	[17] [20] [15] [21]
Low rate of lymph node involvement (despite malignancy)	In histopathological control *** (necessary organ-specific biomarkers and IHC) 14% **(3.7%)**	[16] [19] [22] [23] [24]

All detections were performed using an electrochemiluminescence immunoassay. * Sometimes normal values [given in square brackets] were divergent and apply to individual publications. ** Parathyroid cancer was also associated with moderate to severe hypercalcemia, but with normal calcitonin (CT), thyroglobulin (TG), and high parathyroid hormone (PTH) levels. Mild or encapsulated oncocytic neoplasm of the thyroid was calcitonin-negative, alkaline phosphatase (AP, especially bone fraction) may be useful. *** Most of analyzed papers lacked lymph node biopsy. When writing a diagnostic report, the TNM system was sporadically used with clinical stage and clinical-histological features of nodal micrometastases. TNM staging is not standard. The data presented here were collected from comprehensive reports with a histopathological examination. Recurrence/lymph node involvement by oncocytes were without organ-specific immunohistochemistry (IHC). For differentiation from primary thyroid lymphomas and parathyroid and salivary gland (e.g., Warthin’s tumors), serological biomarker estimation and IHC may be useful (i.e., β2-microglobulin, PTH, serum amylase). Abbreviations: calcitonin (CT); Hürthle cell adenoma/carcinoma (HCA/HCC); immunohistochemistry (IHC); parathyroid hormone (PTH); thyroid-stimulating hormone (TSH); TNM classification of malignant tumors (TNM); total and free tetra-/tri-iodothyronine (TT4/3 and FT4/3).

Precision medicine and the proposed algorithm (Figure 1) indicate the need to examine these elements, in particular to perform TFTs and more detailed imaging tests (e.g., whole-body radioiodine scans (WBS ^131^I), PET ([^18^F]FDG uptake)) before a biopsy is performed. If we do not track TNM staging, assessing the aggressiveness of HCC will be difficult because rapid progression may be the result of late diagnosis, missed (not observed in US) distant metastases, or an extrathyroidal primary oncocytic lesion.

Determination of the thyroglobulin (TG) level was even a greater problem. First, the common presence of anti-TG antibodies gave a number of false-negative results in autoimmunity—most commonly thyroid disease. Second, an increase may be observed in, for example, trauma and radiation damage. However, the presence of a high TG titer may be useful in HCC, contrary to other types (except FTC and HCC, i.e., papillary, anaplastic, medullary cancer, or nodular goiter) [17]. The comparisons of TG level in TC vs. benign and benign vs. healthy indicated a non-significant difference in TG levels (*p*-values were 0.045 and 0.032, respectively), contrary to the comparison of HCC and TC itself [25]. Among 37 patients with HCC, 22 had elevated human serum TG levels. Of note, none of the patients with a normal TG level had a positive whole-body scan [20]. Thus, like other oncological markers, TG level is not useful for diagnosis but for monitoring and may also be valuable in TC differentiation (Figure 2). For example, in immunohistochemistry, a significant serum TG titer allows distinguishing a primary thyroid HCC/HCA from a thyroid metastasis of renal oncocytic cancer [21]. Furthermore, HCC and parathyroid carcinoma patients experience moderate to severe hypercalcemia (as a paraneoplastic syndrome, i.e., without simple bone involvement), but with high TG or PTH level, respectively (Table 1). Of note, HCC is characterized by a high mutational burden affecting the RAS/RAF/MAPK and PIK3CA/AKT/mTOR pathways, an important mediator of many fundamental biological processes, including cellular metabolism, the respiratory chain, proliferation, angiogenesis, and migration [1,26]. Interestingly, the same pathways in plasmacytoma cells promote hypercalcemia by producing Wnt signaling inhibitors (e.g., Dickkpf-1 (DKK1)) to directly suppress osteoblast differentiation with osteolytic bone lesions [27]. Parathyroid hormone-related protein (PTHrP) is a hormone secreted by many neuroendocrine tumors (paraneoplastic phenomenon). In the parathyroid gland, the PTHrP-positive cell level is higher in areas consisting of oxyphil cells. A parathyroid hormone-like effect, characterized by increased renal tubular calcium reabsorption and the inability of the kidneys to excrete the unwanted calcium load, probably contributes to hypercalcemia of malignancy [28]. Furthermore, calcium reabsorption is linked to that of sodium, and low calcitonin increases the serum calcium level, but CT level was only occasionally tested (see below). Therefore, based on the observations summarized in Table 1, calcium homeostasis (Ca^2+^/CT/PTH level) is a crucial factor in the diagnostic chain (initial diagnosis, differentiation, and time-lapse analysis) (Figure 2) and overall survival (OS) (see below in Section 6 Discussion on current methodological limitations).

The second issue is the fact that normal reference ranges for essential TFTs (e.g., FT4, TSH) were usually non-comparable across different laboratories, as shown in Table 1. 

For instance, the reference ranges of the two most common methods in EU, electrochemiluminescence (e.g., ECLIA, manufacturers’ instructions for the Roche 2024-04 diagnostic test and the Cobas PRO/e801 device) and direct chemiluminescence (ALINITY I analyzer, Abbott), were different: TT3—1.3–3.1 (versus 0.35–1.93 nmol/L) and TT4—66.0–181.0 nmol/L (62.69–150.86 nmol/L), respectively. 

Sometimes the TT4 level was described in alternative units (normal 4.87–11.72 µg/dL—ALINITY I analyzer). Moreover, newer studies, such as the one published in 2017 (i.e., after the new classification), contained fragmentary data: TG level without simultaneous examination of thyroglobulin antibodies (common in many thyroid diseases, even in healthy women). The FT3/FT4 or especially TT3/TT4 values were not presented [9]. The euthyroid state was defined imprecisely (usually achieved after thyroxine therapy) [9]. Of note, FT3/FT4 levels reflect hormonal activity, not hormone secretion. This publication was also ambiguous because the disease-free survival of 23.3 years did not correspond to the OS of 20.8 years (OS was determined by contact with patients or members of their families) [9].

The CT level was even less frequently tested using the immunochemiluminescence method, and manufacturers only provide the upper limit of the standard (e.g., <2 pg/mL test Siemens 2018-03 Immulite 2000 Xpi device). Furthermore, there was no standardization and standard ranges varied depending on the method. Ionized calcium levels using the potentiometric method had a range 0.88–1.17 or 1.12–1.32 mmol/L (with various Cobas analyzers). Also, the variation in commercially available TFT results (e.g., FT4) assays led to new specific recommendations [29].

Contrary to data from Poland, the EU, and other countries (Table 1), the study by Li Q. et al. from the Chinese Cancer Hospital described validated reference ranges for TG = 1.28–50 ng/mL (very wide), CT = 0–9.2 pg/mL, and TSH = 0.35–5.1 μIU/mL (chemiluminescence immunoassays using the CL-6000i analyzer) [25]. This is another argument for clinical reproducibility and standardization.

Third, bias is an additional and key problem. Despite the availability of methods, tools, and good practice guidelines, most reports of epidemiologic research incorporated quantitative estimates of bias impact [30]. Even the latest studies and publications (2025) with prognostic significance of selected TFTs (e.g., TSH, FT4, BMI, gender) contained similar methodological shortcomings [31]: (1) only retrospective data, usually collected in the twentieth century, (2) no control group, i.e., patients with similar TFTs but with benign lesions or healthy, (3) misnomers: interchanging TC (in title) and DTC (methods), (4) did not implement the TC imaging guidelines of the 3rd or 4th edition of the WHO Classification of Thyroid Cancers. In particular, HCC was not included.

Such an observation indicates that these parameters are useful for monitoring patients with already diagnosed cancer (Figure 2B), not for preliminary diagnosis (Figure 2A). Therefore, in this context, these tests do not have a significant advantage over tests for thyroglobulin or other tumor markers. Furthermore, few studies included a control group, illustrating the probability of false-positive TC markers. However, this methodology requires changing habits, i.e., collecting data before and after therapeutic interventions [17,25]. In this isolated study, the repertoire of diagnostic tests was narrow (e.g., TSH, CT, CEA). Interestingly, a statistical relationship with TC and a benign tumor process was completely excluded (*p*-value close to 1) when analyzing general history (e.g., family genetic history, coexisting metabolic syndrome, radiation exposure) [25]. However, this does not resolve the issue of the involvement of metabolic changes in HCC, because (as in other studies) the relationship with TC itself was examined. This metabolic variation in tumors seems to be crucial, especially in HCC as an example of oncocytic tumors, since cysteine accumulation is not observed in these malignancies. Equally important clinicohistological features, such as compression symptoms, were not observed, nor regional perfusion defects related to compression phenomena nor deafferentation [32]. Furthermore, when the oral glucose tolerance test (OGTT) was performed, the 120 min glucose concentration was 9.1 (normal < 7.8 mmol/L), with impaired glucose tolerance (IGT) and no suppression of growth hormone (GH). Therefore, IGT, i.e., reduced tissue glucose uptake, may influence PET findings (hypermetabolic activity—see below) due to a paraneoplastic manifestation and increase mortality due to HCC if insulin-like growth factor 1 (IGF-1) is elevated to 29 (normal, 5.8–25.5 nmol/L) [15]. Of note, transcriptional control of thyroid transcription factor (TTF-2) and TG by IGF-1 were described [33,34]. 

In our comprehensive analysis, many observations (if not the majority of them) of TFTs and risk factors for cancer, thyroiditis, and adenoma were retrospective [8,9,11,14,25], but an underrated problem in data collection is the fact that commercial laboratories and health insurance companies include selected and cheap tests (usually only TSH or FT4 and serum anti-thyroglobulin antibody (anti-TG), which cost about 2 or 4 EUR, respectively) in various “women’s health checkups”, anthropological research, or occupational medicine [35]. This has a significant impact on further diagnostics, FNAB, and surgery, since in one study from Poland with 1078 consecutive patients who underwent thyroidectomy, the female to male ratio was 9:1 [36].

Consistent with a previous report [37], we recommend further research and re-evaluation for understanding the endocrine pathway (e.g., pituitary–hypothalamic–thyroid axis) in HCC due to non-comparable data and non-standardized, narrow TFT panels in several recommendations.

### 3.2. Medical Imaging

#### 3.2.1. Ultrasonography (US)

Outstanding, safe, and confident diagnostic imaging is the foremost objective of any radiology practice. Although US is the most common medical imaging method, there is no universal and simple assessment algorithm. Many regional and local scientific societies (including endocrine, radiological, and oncological groups) develop their own guidelines. Most algorithms are used to qualify for FNAB, but they are not conclusive. Importantly, such algorithms—which are increasingly complex—do not serve to assess other disease entities that are much more common than TC, such as Hashimoto’s disease [38]. 

Mild hypoechogenicity was significantly less related to malignancy (odds ratio [OR]: 1.409; CI: 1.086–1.829; *p* = 0.01) compared to moderate (OR: 4.775; CI: 3.700–6.163; *p* < 0.001) and marked hypoechogenicity (OR: 8.540; CI: 6.355–11.445; *p* < 0.001) [39].

The Thyroid Imaging Reporting and Data System (TIRADS) is time-consuming and most observations are largely based on suspicious ultrasound features derived from PTC (e.g., marked hypoechogenicity, spiculated margins, lymph node metastasis) or FTC [8]. Furthermore, a mandatory Doppler involving thyroid parenchyma blood flow intensity and peak systolic blood velocity of thyroid arteries, which indicates the magnitude of neurovegetative influence, has been suggested [39].

Bias and unnecessary thyroid ultrasound examinations in the asymptomatic general population were described [40]. Since the prevalence of Hashimoto’s disease worldwide is about 0.3–1.5 cases per 1000 people per year and it is much more common in women, (the ratio varies in publications from 5:1 to 20:1), these facts affect the frequency of follow-up visits and ultrasound examinations to assess nodules and thus the TC rate (most reports are retrospective studies) [41]. In our observation, in Poland, US is performed particularly frequently in women motivated by a “health checkup” or aesthetic medical reasons (author’s own unpublished data) [41,42,43].

#### 3.2.2. Scintigraphic Imaging

^18^F-fluorodeoxyglucose positron emission tomography (PET) combined with computed tomography (FDG-PET/CT) has been widely used in oncology. It may be used for the localization, staging, prognosis, and follow-up of HCC because the normal thyroid gland shows very low-grade [^18^F]FDG uptake [44]. It is a good alternative for whole-body scans after 200 mCI ^131^I treatment to show thyroid involvement in HCC [45]. Unfortunately, a discrepancy exists due to low HCC radioiodine avidity (see section about radioactive ^131^I treatment). Furthermore, other scintigraphic techniques based on technetium-99m isotopes are a promising alternative, since 99mTc is selectively concentrated in the stomach, thyroid, and salivary glands. Double-phase scintigraphy with technetium-99m-sestamibi may be a useful technique in HCC [46], but parathyroid cancer is also possible [47]. Furthermore, 99mTc is excluded from cerebrospinal fluid, but when 99mTc is chemically bound to the carrier (e.g., combined with perchlorate), its selectiveness is abolished. Unfortunately, 99mTc scan was described to be unreliable in detecting brain metastases from HCC due to the lack of 99mTc uptake in metastatic lesions [32]. 99mTc pertechnetate scans showed an enlarged gland with increased uptake of radiocontrast in patients with Hashimoto’s thyroiditis (HT) and Graves’ disease (GD) [48].

The conservative paradigm is that, in [^18^F]FDG PET/CT scans, high risk of malignancy coexists with high metabolic activity and high standardized uptake value (SUV). Likewise, oncocytic thyroid nodules with focal FDG avidity were misinterpreted as having a higher potential of being malignant (see below) [49]. Oncocytes were characterized by intense metabolic activity and high glucose uptake (visualized by FDG PET/CT scan). This can be explained by the abundance of intra-cytoplasmic mitochondria [50]. Metabolic activity (glucose catabolism) is one thing; proliferation is another. High mitochondrial content and mutations in respiratory chain genes indicate that with little proliferation, metabolic symptoms may dominate. The OGTT and assays measuring the entire event of cell activation are more sensitive than classical tritiated thymidine intake (observed in S-phase). The method resembles PET and reveals that the proliferative activity of cells is primarily metabolic, as described previously for the glucose consumption test (an exponential curve depicts the rapid decrease in glucose level during mitosis) [51]. Furthermore, in HCC, patient IGT (i.e., low glucose influx in peripheral tissues—see Section 3.1 Laboratory data) can reduce background activity and intensify contrast in PET.

Evaluation of the clinical significance of PET in differentiated thyroid carcinoma and comparison of the results with other imaging modalities (e.g., whole-body ^131^iodine scintigraphy (WBS), technetium-99m-hexakis-2-methoxyisobutylisonitrile (I) scintigraphy (MIBI)) were described [52]. To our knowledge, no study has specifically focused on [^18^F]FDG-PET/CT for diagnostic purposes in HCC, since the same problem was described previously for FTC [8]. 

Finally, we need to leapfrog, especially when it comes to lymph node involvement and imaging efficiency, since most of the papers analyzed were without lymph node biopsy (control) and the data presented in Table 1 and Table 2 were obtained from comprehensive case reports with a histopathological examination. This confirms our previous observation, where TNM (feature N) turned out to be difficult (metastases to lungs through the blood system dominate, sometimes diagnosed as lung cancer) [53].

Surveillance with standard for DTC tools (e.g., TSH stimulated diagnostic whole-body radioiodine scans (DxWBS), TG levels) is not adequate for HCC (low radioactive iodine uptake, TSH receptor-negative cells) [20,57,58].

Of note, despite the well-known paradigm in TC (the so-called “flip-flop” phenomenon [58]), well-differentiated HCC shows greater [^18^F]FDG than ^131^I uptake in scintigraphy. In this observation, such “reverse flip-flop” is observed in HCC due to (1) low radioiodine avidity and low TT4 production (see Table 1); (2) high catabolic (mitochondrial) activity of HCC due to the Warburg effect; (3) shift to aerobic metabolism; and (4) paraneoplastic phenomena, i.e., RAS/RAF/MAPK and PIK3CA/AKT/mTOR pathways [26] with calcium signal (Table 1, see Section 3.1).

### 3.3. First Step and Mispathology

Besides variations in terminology, a high degree of intraobserver variability was demonstrated in the interpretation of FNAB thyroid specimens. Simple second-round review of all thyroid aspirates was described as impractical [59]. In the last single-center analysis, which just guaranteed greater comparability (similar approach and technique, the same team of histopathologists), positive predictive FNAB value was satisfactory in follicular TC (53%), but very low in HCC (15%) [60]. This corresponds with our observation. The same lessons were drawn from the use of gene expression classifier: most samples were follicular TC (FTC) with non-oncocytic features [60]. Consequently, a small number of cases was the first crucial limitation for assessing diagnostic accuracy [61]. Of note, although the study was conducted at a large academic medical center, the “oncocytic features group” (as sometimes used in the literature as well as in guidelines) consisted only of three Hürthle cell adenoma cases [62]. This proves how rare our observation is and the generalization resulting from collective terms (e.g., DTC, FTC, “oncocytic feature”) develops inconsistencies and inadequate data, such as statistics, meta-analyses, and guidelines. Oxyphilic cells may be seen in very rare metastatic cancers. For example, renal cell carcinoma metastasis to the thyroid is the potential scenario, but this is not true HCC because it is not derived from the thyroid follicular epithelium. Unfortunately, head and neck neoplasms with oncocytes in the thyroid gland were also found (Table 1 and Table 2) [63]. Furthermore, mitochondrial accumulation as well as mitochondrial DNA expression were unique effects unrelated to genetic cell cycle regulation, resulting in whole-cell abnormal proliferation (mitosis with S-phase). HCC with uncommon genetic, endocrine, and metabolic background was a unique type of TC that was relatively poorly described in the literature and its classification is in the development stage.

In comparison to clinical data, histopathological statistics were less conclusive about the malignancy and aggressiveness of HCC, often containing a number of contradictions and methodological inaccuracies. Narrow insight due to overuse of fine-needle aspiration biopsy (FNAB) and balancing the radicalness of a local surgery with the preservation of aesthetic and functional outcomes are good examples of different approaches in various scientific and clinical centers or by individual doctors (e.g., endocrinologist, otolaryngologists, oncologists, head and neck surgeons) [64]. The decision about FNAB is usually based on ultrasound examination (US) in the diagnosis of goiter (Table 2). The most common clinical presentation of follicular thyroid cancer was a single, painless thyroid nodule. US findings of oncocytic/Hürthle cell neoplasms were solid content, oval to round shape, hypoechogenicity, a smooth margin, the halo sign, and no calcification (despite significant hypercalcemia), but tumor size was only described as a predictor of malignancy [65]. In the immunoendocrine background of HCA/HCC, chronic lymphocytic (Hashimoto) thyroiditis may have multiple oncocytic lesions or oncocytic changes. In Hashimoto’s disease, FNAB often reveals reactive and polymorphous lymphoid cells and occasional epithelial cells with Hürthle cell changes [66]. Therefore, like all methods, FNAB also has its advantages and disadvantages (Table 2). At this stage, scintigraphy and nuclear medicine techniques (see Section 3.2.2. *Scintigraphic Imaging*) are rarely used for practical and economic reasons. Although HCC is well described, the 5th edition of the WHO Classification of Endocrine Neoplasms introduced arbitrary oncocyte proportion (75%) as a crucial criterion [4]. This criterion significantly reduces the number of diagnosed HCCs, and existing HCC statistics, as well as FNAB-based data on thyroid cancer, should be verified with caution. Oncocytes are also seen in thyroid neoplastic diseases, both benign and malignant, including, among others, variants of papillary TC and the oncocytic variant of medullary TC. The critical review by Asa S.L. demonstrated oncocytic thyroid neoplasms, i.e., papillary and medullary cancer with oncocytic features [67]. This new insight calls attention to the critical role of accurate immunohistochemistry in TC pathology for quantifying cellular proliferation, including assessing mitotic activity and Ki-67 expression [68]. Furthermore, cytomorphological HCC misfindings were described in parathyroid cancer [54], which confirms the need for calcium level tests and further tests proposed above (Section 3.1, Figure 2, Table 1), especially in the case of lymph node involvement (Table 1). On the other hand, coexistence of HCC and parathyroid carcinoma were also described [69].

Although the diagnosis of an oncocytic tumor is not usually difficult due to its distinctive features, in borderline situations, mitochondrial markers can be used, including antimitochondrial antibodies (113-1) [70]. However, antimitochondrial antibodies are not organ specific and are usually observed in autoimmune thyroiditis (20.4% patients had Hashimoto’s thyroiditis; 3.2% had Graves’ disease) [71]; thus, mitochondrial markers may provide false-negative results with very low predictive FNAB value [60]. Such an aggressive immune response is considered one of the sources of oncogenesis, e.g., in FTC (cellular cytotoxicity), especially thyroid lymphoma (lymphoproliferative disease).

Not all oxyphilic cells are true oncocytes and not every aspirate containing a large amount of oncocytes (i.e., 50%) is appropriate for analysis: FNAB molecular tests may be performed afterward [72]. For example, from 172 patients with thyroid nodules, 39 presented with cytologic findings of oncocytic changes on FNAB (suspicious for HCC), but finally after surgery only 2 cases (about 5%) were true HCC [73]. Therefore, it is hard to discern the significance of oncocytic changes in FNAB reports and, for the surgeon, to determine an optimal approach [74]. The proverb “easy come–easy go” for cytology-based techniques (e.g., FNAB, EBUS, EUS) suggests a general rule that every in-depth and complementary diagnosis costs a lot of exertion and has its complications [54,75].

Second, due to embryogenesis (see below) in the immediate surrounding areas of the salivary, thyroid, and parathyroid glands, there is no clear answer whether the oncocytes observed in the thyroid gland actually come from follicular cells or are metastases. Narrow specialization in oncology centers is an obstacle here, and depending on the unit, the lymph node (hematology unit), the salivary gland (laryngology), or the thyroid gland (endocrinology) will be collected first by FNAB, but the parathyroid glands are never collected.

Collective arguments (presented in Figure 2) point to the fact that FNAB is not a suitable tool in HCC for the following reasons (Table 2 and Table 3): (1) FNAB is the cause of misdiagnosis of Hürthle cell neoplasm, and lesional cells are frequently miscategorized as Hürthle cells or oncocytes more frequently than granular cell tumors [75]; (2) lymph node metastasis is not examined; (3) the coexistence of oncocytes and other cells makes assessment of strict proportions difficult, contrary to a decisive factor (75%) according to the 5th edition of the WHO Classification of Endocrine Neoplasms; (4) usually low cellularity (the immunohistochemistry and genetic background not performed); (5) the malignancy criteria (e.g., capsular, vascular invasion) are impossible to assess; and (6) in FNAB, HCC may represent metastatic oncocytes.

On the contrary, core biopsy and total thyroidectomy are offered as ambulatory procedures [81]. The biggest study group in Poland showed that most oncocytic tumors were benign, but strict histopathological diagnosis of HCC/HCA differentiation was very difficult (see section about malignancy). Intraoperative pathological diagnosis often lacks the discriminative power of fast and accurate histopathological examination [77].

## 4. Translational Medicine: HCC Pathology and Presentation

### 4.1. Oncocyte and HCC Origin (Cancer Stem Cells?)

The dogma of cancer biology is the concept that the phenotype of tumor cells necessarily reflects their origin from specific progenitor cells. However, it is expected that such tumors with dual differentiation will reflect the nature of their progenitor cells. In the thyroid, there may be stem cells that have the dual potential to differentiate toward follicular cells and C cells.

Within head and neck cancers (HNC), oncocytic cells are described in acinic cell carcinoma, and Warthin’s tumor is extremely difficult to differentiate [76,82]. The WHO classification of salivary gland neoplasms recognizes three oncocytic entities: oncocytosis, oncocytoma, and oncocytic carcinoma. Of note, in the embryonic development of vertebrates, the second pharyngeal pouch gives rise to salivary glands and tonsils [83]. Pharyngeal pouches develop on the endodermal surface between the pharyngeal arches, but pharyngeal grooves (clefts) form on the ectodermal side of the neck region to separate the arches. The thyroid gland also develops from the pharyngeal endoderm at the level of the second arch, but it originates medially from the endodermal tissue in contact with the aortic sac. Over the course of embryogenesis, all of the arch components differentiate into distinct derivatives [84], but the endoderm of the pharyngeal arch forms the pharynx epithelium and glands [85]. These correspond to the new WHO TC classification of salivary gland-type tumors (e.g., mucoepidermoid carcinoma, secretory carcinoma), now included in one section classified as “salivary gland-type carcinomas of the thyroid.” 

Parathyroid glands adjacent to the thyroid gland, which also contain oncocytes [76,82], come from the third pouch [85,86]. Thyroid oncocytes (Hürthle cells) originate from a median endodermal mass in the region of the tongue (foramen cecum), and early stages of development of the thyroid are similar to those of the third pharyngeal pouch derived from the salivary and parathyroid glands, with the formation of a bud like structure from the endoderm [87]. In our opinion, this may be an argument for valid changes in the 5th edition of the WHO Classification of Endocrine and Neuroendocrine Tumors. Hürthle cells are excluded as a misnomer (see Section 3. *Oncocytic Thyroid Cancer Diagnosis—Standardization as the Highest Scientific and Clinical Need: Precision Medicine*).

Interestingly, according to the human gene atlas, TG genes may be expressed in the heart and tonsils, and more precise immunohistochemical staining with detectable thyroglobulin is 100% specific for thyroid tumor origin [88]. Although the last publication stated that the presence of amylase crystalloids is not a specific marker of salivary gland pathology [89], more specific observation of nontyrosine eosinophilic color crystalloids was described (e.g., in Warthin’s tumors) [90]. This suggests (as in immunohistochemistry) that analysis of serum levels of calcium, amylase, PTH, and TG (Table 1, Figure 2) may be useful in oncocytic tumor differentiation, although highly dedifferentiated oncocytic cells may be negative for these markers. Lack of differentiation (here, TG, PTH, CT, and amylase-negative oncocytes) is considered a hallmark of aggressive malignancies. In this respect, other markers may also be useful for assessing differentiation and aggressiveness, since the thyroid normally contains neuroendocrine (NE) cells and it is not surprising to find NE differentiation in HCC.

TC’s ability to produce a variety of peptide hormones and related substances, such as CT, ACTH, and α-hCG, was proved by chemical assays or immunohistochemical techniques. Oncocytes in HCC were reported to be immunoreactive for NE markers such as neuron-specific enolase (NSE), synaptophysin, chromogranin A, and serotonin [91]. 

In the first report of immunoreactive cases detailing three markers (NSE, neuron-specific enolase; Syn, synaptophysin and calcitonin), 5 were identified as papillary and 1 was identified as Hürthle cell carcinoma [92]. However, a very rare oncocytic variant of medullary thyroid carcinoma may be a mimicker. Its cytomorphology overlaps with more common Hürthle cell lesions [92]. On the other hand, the presence of NE markers may indicate a common origin of HCC and C cells from endodermally derived ultimobranchial stem cells or crest cells [88]. Common stem cell origin from neural crest is favored for tumors of such dual differentiation, but the origin of oncocytes remains an unresolved question. Contrary to the oncocytic variant of medullary carcinoma, neuroendocrine oncocytes (from mild HCA) were CT-negative and, noteworthy, no mitotic activity was found [18]. This does not change the fact that such neuroendocrine activity and expression of CD56 (neural cell adhesion molecule) play crucial roles in paraneoplastic syndrome, quality of life, as well as HCC malignancy (see below).

A correct initial diagnosis by fine-needle aspiration and immunohistochemical panel (e.g., synaptophysin, chromogranin, NSE, CD56, CT) is imperative for the clinical treatment for HCC. In our opinion, in most immunocytological studies, multicolor flow cytometry is warranted (e.g., with antibody and neuroendocrine markers proposed by Filotico M. and Plutino F. [18]).

### 4.2. HCC Etiology

The influence of various genetic, environmental, and biological factors is observed in HCC pathogenesis. Oncocytes are epithelial cells with abundant, granular, eosinophilic cytoplasm, which results from multiple mitochondria. Independent of cell division (given by nuclear gene mutation), they are strongly associated with alterations in the complex I genes of the mitochondrial respiratory chain [1,26,93]. Therefore, such cells are not typically observed in HCC only but preferentially in glands (e.g., thyroid, parathyroid, salivary glands, pancreas, adrenal cortex) with significant genetic background, i.e., deletions/rearrangements in mitochondrial DNA (of note, maternal), as reviewed by Maximo V et al. [93]. However, oncocytic lesions in renal tumors should be considered a different category since no such genetic changes have been recorded and cells are not of glandular origin [94]. Notably, cell proliferation rate and tumor mass are not the main factors of mortality in HCC and neuroendocrine tumors; instead, paraneoplastic phenomena, secondary infections, and metabolic disorders, such as cachexia, are usually the primary drivers (see below Section 4.3) [53,95].

However, cited epidemiological data were often very limited because HCC was recently distinguished as a separate type. Other causes of mortality were not taken into account when assessing five-year survival [96]. Multiple endocrine neoplasia type (MEN) was established as a risk factor for various thyroid malignancies, including oncocytic carcinoma [97], but the MEN2 phenotype involves high risk for the development of NE medullary carcinoma [98], while the MEN1 phenotype involves two or more endocrine tumors, including parathyroid, anterior pituitary, and/or gastroenteropancreatic tract tumors. Notably, the 5th edition of the WHO Classification of Endocrine Neoplasms introduced a more detailed cytogenesis-based classification (e.g., pituitary neuroendocrine tumors) as well as the reclassification of thyroid neoplasms, based on cytogenesis and pathogenesis. Furthermore, multiglandular disease (for example, in primary hyperparathyroidism) was described as multiple adenomas in genetic tumor syndromes [4,99]. 

Moreover, exposure to ionizing radiation, described in a number of publications, is not a real factor. Neither reactor accidents nor radiotherapy are a factor in the USA today, but HCC accounts for about 2% of TCs (i.e., 880 cases per year) [96]. This review discussed exposure to ionizing radiation as a common risk factor for thyroid cancer overall, without considering metabolic changes such as the massive use of GLP-1 (Ozempic) [100]. Different conclusions were reached by the Polish Cancer Registry. In comparing the last decade of the 20th century (to the end of 1999) and the second decade of the 21st century (the last period), there were far fewer cases than today (1357 vs. 2879), but the share was similar (1.18% and 1.23% of cancers), as was the number of deaths due to TC (279 vs. 319) and the cumulative risk (0.34 vs. 0.32). Significant differences in TC incidence are therefore a result of recognition (like other cancers) over the years (availability of diagnostics), since the first period could have been influenced by accidents at nuclear plants near Poland and the TC prevalence was significantly lower (36,761 vs. 73,262 patients) [101].

The same misclassification is observed since severe (sometimes fatal) thyroiditis with oncocytes in FNAB may be classified as mild or malignant, i.e., oncocytoma or oncocytic carcinoma (see below), and approximately one-fourth of people worldwide have thyroid nodules, but only 10% to 15% of these nodules are malignant [102]. Contrary to strict FTC classification (follicular adenomas vs. follicular thyroid carcinomas), the same issue was not considered previously for HCC [103]. 

Although a grading system for medullary TC was also introduced (based on mitotic count, tumor necrosis, and Ki67 labeling index) the same issue (i.e., grading and malignancy sign) was not discussed for HCC. In disease-free survival (DFS), analysis was not available for Ki-67 as an independent risk factor in HCC [8].

### 4.3. Oncocytic Malignancy—HCC/HCA Differentiation

Malignancy in cancers is characterized by anaplasia, invasiveness, and metastasis. Historically, it was believed that an accurate differential diagnosis could be made between cancer and adenoma on the basis of pathological studies: oncocytic adenomas had primarily a follicular structure and trabecular, solid areas. Furthermore, a false paradigm was that the criteria for distinguishing between benign and malignant oncocytic neoplasms were not different from those used in the diagnosis of ordinary follicular tumors [104]. This is a crucial argument against FNAB in HCC diagnosis (Table 3). Second, differentiating oncocyte cells by morphology as malignant is very difficult: for example, oncocytic variants of medullary carcinoma vs. HCC [78]. Therefore, it is a flawed paradigm that preoperative FNAB remains the most accurate diagnostic method for detecting thyroid cancer [105].

HCC can be hardly distinguished from HCA in a simple pathological study, especially cytopathology. However, contrary to other TCs with nuclear DNA mutation (especially anaplastic TC), oncocytes in HCC are characterized by mitochondrial mutations and a high degree of differentiation [79]. Thus, anaplasia and lack of differentiation, considered a hallmark of aggressive malignancies, are not clear in HCC. Nuclear pleomorphism/atypia (a property of Hürthle cells), multinucleation, high nuclear-cytoplasmic ratio, and high proliferation index (mitotic activity) may be absent and were not considered useful for predicting prognosis [67]. Invasiveness may be observed in two different modes—macroscopic (clinical imaging) or microscopic vascular/lymph node invasion after surgical/core biopsy—but not in FNAB (Table 1 and Table 3). Oncocytes occurring on a background of Hashimoto’s thyroiditis or nodular goiter cannot be distinguished either morphologically or histochemically from adenomatous or carcinomatous Hürthle cells. Histologically, the high malignant potential of HCA remains very controversial [79]. 

For example, among 846 patients with thyroid enlargement, only 62 (7.3%) had a confirmed malignancy. Among malignancies, papillary thyroid carcinoma was the leading one (28 cases, 45.2%), followed by follicular thyroid carcinoma (18 cases, 29%), and the least observed types of thyroid malignancies were medullary thyroid carcinoma and Hürthle cell carcinoma, each accounted for one case [106]. Therefore, 1.6% of TCs were HCC, but only 0.11% (1/846) of patients had thyroid enlargement (most of them showed autoimmune thyroiditis with oncocytic background).

#### 4.3.1. Clinical Sign of HCC Malignancy

Data from various patients and situations (diagnostic procedures, not only therapeutic ones) revealed that malignancy was diagnosed in follicular and Hürthle cell neoplasm in 143/428 (33%) and 64/309 (20%) of cases, respectively, which indicates that oncocytic tumors have a lower tendency toward malignancy [23]. In this respect, in the literature we can find reports and macroscopic evidence of high aggressiveness (malignancy) of HCC much more frequently [32,107,108]. The metabolic and neuroendocrine properties of HCC seem to be crucial. Numerous case reports with oncocytes in brain metastasis suggested that HCC/HCN was a dangerous/serious condition, but without clear HCC anaplasia (clinical vs. pathological sign of malignancy). Of course, in such cases of metastasectomy without HCC confirmation, oncocytes from the kidney or other locations (e.g., salivary gland) or multiorgan Birt–Hogg–Dubé (BHD) syndrome cannot be ruled out [7]. BHD is an autosomal dominant genetic disorder characterized by small papular skin lesions (fibrofolliculomas) causing susceptibility to kidney cancer, renal and pulmonary cysts, spontaneous pneumothoraces, and several noncutaneous tumors [7].

#### 4.3.2. Lymph Node Involvement

In common practice, attention is focused on assessment of the thyroid gland. Usually, assessment of other structures of the head and neck (e.g., regional lymph nodes, parathyroid glands, salivary glands) is omitted [61]. As presented in Table 1, lymph node involvement was reported sporadically. In spite of extensive capsular and vascular invasion, no lymph nodes were occupied [16]. A recurrence in regional lymph nodes was diagnosed in 3% of patients [109]. Another case of advanced angioinvasive HCC was also described without lymph node involvement [19]. In a single-center observation, despite the large tumor size (median 6.7 cm), only one patient showed positive lymph node involvement. Notably, the median time to death was the shortest (4 years), contrary to distant metastasis (13 years) and locoregional recurrence (6 years) [110]. 

These representative publications revealed that histopathological evaluation of regional nodes was not practiced. For example, among 277 patients, only 190 had a total thyroidectomy (TT) [111]. Of course, the statistics were higher (up to 14% of those evaluated), but they were based only on a small percentage of patients whose tumor size was large enough for nodal evaluation to be performed (vascular invasion was not surprising). Conversely, in a large study of 12,438 patients with HCC, only 432 (3.7%) had proven pathologic nodal metastases [24]. In this respect, PET-CT data may be more reliable and helpful (Table 2, Figure 2), but this is not a common practice in TC. 

One quite critical study showed some discrepancies: the rate of pre-operative diagnosis of well-differentiated thyroid carcinomas in this unit was under 50%, FNAB was inadequate in 11 cases (10%), and 10 patients had surgery for benign disease. Among 109 patients, 38 had a definite pre-operative diagnosis. In 61 cases, a malignant tumor was suspected. The findings indicated a benign lesion in 47 patients, a suspicious lesion in 13 patients, and a malignant lesion in 38 patients diagnosed with thyroid carcinoma [80]. Similar observations came from Poland, including the author’s personal experiences.

## 5. HCC Therapy

Controversies still exist around the management of Hürthle cell tumors, and the paradigm is treatment based on surgery. On the other hand, there is still controversy concerning management of mild oncocytic tumors, because HCC can be hardly distinguished from HCA [79].

Initially, before any procedures, all diagnostic methods should be used as a reference point in the diagnostic chain (Figure 2) because the effects of surgical procedures and their choice for HCC have not been evaluated. The presence of bilateral nodular disease and the patient’s comorbidities should be considered when deciding on the extent of surgery. However, the complexity of the therapeutic regimen (neoadjuvant or adjuvant therapy) depends on whether the procedure is therapeutic only (after FNAB) or a diagnostic–therapeutic algorithm. Since differences between Hürthle cell adenomas and Hürthle cell carcinomas could be clearly made only by histopathological evaluation and a number of listed arguments (see above, Table 1, Table 2 and Table 3), the first method should not be continued. Bias and misrepresenting statistics (conflicting data) constitute an additional argument, because FTC outcome was analyzed only in retrospective observational studies [8].

Of note, often after radical surgery, treatment is supplemented by postoperative endocrine therapy (different thyroxine supplementation), patient evaluation is not continued: scrupulous diagnostic tests (e.g., immunohistochemistry, metabolic and immunoendocrine background) are usually discontinued despite the coexistence of systemic complications. 

However, intraoperative tests should be further considered prior to basic decisions regarding removal or not of the entire thyroid gland (HCC preferential method), surrounding lymph nodes, and remnant tissue, scrupulous diagnostic tests (e.g., immunohistochemistry, molecular background), and identification of loco-regional recurrent disease [112].

After final histopathology, surgical retreatment may be necessary [56]. Of course, the radicality of the procedure was assessed in very different ways, based mainly on the margin of healthy tissue and invasion of vessels and capsule. These systems were largely based on statistic features derived from FTC/PTC, not HCC [109].

### 5.1. Treatment with Radioiodine Therapy (RIA)

In one comprehensive study, patients who received pre-operative 99mTc and/or ^131^I thyroid scan showed cold nodules. Of note, only one carcinoma patient developed neck lymph node metastasis (with normal serum thyroglobulin, negative ^131^I but positive 99mTc-MIBI whole-body scans) and another one showed mediastinal metastasis (with elevated thyroglobulin and positive ^131^I uptake) and revealed successful regression after ^131^I therapeutic regimen [55]. Therefore, radioiodine ablation is indicated whenever there is ^131^I uptake by tumors in initial, preoperative examination in the diagnostic chain (currently out of practice) (Figure 2).

The data presented in Table 1 and Table 2 (e.g., low TT4 production, low radioiodine-avidity) indicate that RIA in HCC may be not effective.

Well-differentiated thyroid cancer (WDTC) is characterized by favorable disease course and excellent survival, but the collective term is a misnomer, which forces further therapeutic errors, such as postoperative radioactive iodine therapy [113] Furthermore, TC treated with ^131^I may provoke serious neurological problems [31,32]. After high activity treatment, many patients suffered grade 4 WHO hematological toxicity with an important decrease in qualify of life. Significant salivary gland morbidity was observed (30% dry mouth, 27% salivary swelling); therefore, OS and the risk–benefit ratio are controversial [114].

### 5.2. Thyroid-Stimulating Hormone Suppression Therapy (TSH-ST) Overuse

In patients with high risk of follicular or papillary cancer, initial treatment with doses of levothyroxine may be sufficient to suppress thyroid-stimulating hormone levels (i.e., TSH-ST). However, if we look at cancers with a genetic background (e.g., BRAF mutation), despite various localizations, thyroid hormone overuse may be the cause of secondary oncogenesis, as reviewed in ref. [115,116]. This challenges the known paradigm of suppressing the growth of thyroid cancer per se. In most publications, <0.1 mU/L TSH suppression was proven to be beneficial for patients likely to have microscopic or macroscopic disease, as TSH had a direct trophic effect on thyroid cancer cells without strict TC type [117].

First, a collective term of differentiated thyroid cancer (DTC) is used. Furthermore, because DTC is the most common group of TCs in the medical literature, sometimes titles of publications generalize the collective term, and the terms thyroid cancer (TC) and DTC are interchangeable [118].

Second, contrary to the known paradigm, DTC in patients with Graves’ disease was described as more aggressive. A low TSH level (as in Graves’ disease) can be either a good or bad prognostic marker, depending on whether TC is rated qualitatively (positive or negative biopsy after surgery) or quantitatively (tumor size). Furthermore, the statistical relationship is not a strict pathway and cause–effect link. Papillary and follicular carcinomas together are called DTC due to their histologic resemblance to normal thyroid tissue and differentiated function (e.g., secretion of thyroxin and thyroglobulin (TG)). Thus, native thyroxin (T4) and TG are used for evaluating patients after near-total or total thyroidectomy (with or without radioiodine ablation (RIA)) for DTC only. Normal or elevated T3/TG values (Table 1) indicate the presence of residual normal or cancerous thyroid tissue with differentiated function [119]. Unfortunately, this does not apply when the patient undergoes hormonal treatment (see Section 3.1) [9].

Third, the target of medical therapy may be the patient’s life or reduction of the disease, with various risk–benefit ratios. It is quite different if we use the overall survival (OS) (i.e., patient-centered care (PCC), precision medicine) versus the risk of relapse/progression free survival (PFS) as a measure (i.e., disease-directed therapy, DDT). Excessive thyroid hormone replacement can lead to atrial fibrillation, cachexia, and several multiorgan complications [120], and optimal TSH levels are inconclusive. The crucial question is: What is the purpose of medical intervention? Endocrine results (low TSH level) [121])? Oncology (PFS)? Or holistic medicine (OS with good quality of life (QoL)) [37,121]?

Of note, the PFS laboratory results, as well as the patient’s opinion, are the surrogate measures of clinical benefit and the patient’s general activity and performance (expressed, for example, as the Karnofsky performance score). Benefits need to be weighed against the risks with good holistic attempt and full understanding of the mechanisms by which survival is extended. The mechanism of the action of a drug, specific manifestation of the disease, and homologous population (e.g., strict TC type) under study should be analyzed [122]. Contrary to various cancers, endocrine neoplasms, as a general rule, show systemic metabolic (IGT), immunoendocrine (autoimmune background), and paraneoplastic (hypercalcemia, “reverse flip-flop” phenomenon in HCC) consequences despite small tumor size, without node involvement and metastasis (TNM) (Table 1).

According to current multidisciplinary guidelines, TSH-ST is described as “not necessary” without distinguishing two different situations: TSH-ST versus T4-ST [123]. 

## 6. Discussion on Current Methodological Limitations: Bias, Non-Standardized Nomenclature (Miscategorization), and Diagnostic Chain (Misdiagnosis)

In our work, we pointed out a number of methodological difficulties and inaccuracies. Most studies did not have sufficiently described methodology, such as standardized TFTs, lack of IHC, and differentiation [79]. The main obstacle seems to be the specific language used in the literature. Follicular cell-derived carcinomas are divided into papillary (PTC), follicular (FTC), oncocytic (HCC), poorly differentiated (PDTC), and anaplastic thyroid carcinoma (ATC). PTC, FTC, and HCC are considered well-differentiated thyroid carcinomas [118,119]. Differentiated thyroid cancer (DTC), which includes predominantly papillary and follicular thyroid cancers (in many publications without HCC), operates as a convenient term. Studies in which patients with HCC were analyzed as a group with other thyroid carcinoma types were biased (miscategorization—see graphical abstract) [8]. As a rule, in cohort studies, to gain a high number of patients and professional impact (and citations), collective terms (DTC or TC) and introducing the topic in broad terms are preferred in the literature, especially in meta-analyses [14,120]. For example, despite the PRISMA (Preferred Reporting Items for Systematic Review and Meta-Analyses) statement, the terms TC (in the methods section ,i.e., search process) and DTC (in the results section) were used arbitrarily [120]. Incorrectly, these two keywords were used alternately or simply by accident. The search strategy and selection criteria described in many reviews conflicted with real practice, since the term “Hürthle cell carcinoma” was in the title, but most data (e.g., tumor size, Ki-67, histological subtype (widely invasive vs. minimally invasive)) were presented for FTC only or collectively (for FTC + HCC) [8]. Incidence data were obtained from populations (22,738 patients from SEER 9 registries) offering non-complete data for HCC because microFTC and microHCC were combined into a single group (mFHCC) [124]. Therefore, in such studies, patients with HCC were analyzed as a group with other TCs [22]. Of note, till 2017 and the 4th edition of the WHO classification, Hürthle cell adenomas and carcinomas had been regarded as variants of follicular neoplasms; therefore, statistical data obtained from any pre-existing registries contained this key limitation. However, numerous meta-analyses based on retrospective data seemed more up to date. Furthermore, the 5th edition of the WHO Classification of Endocrine Neoplasms introduced a new quality in the field (75% oncocytic cell criterion) and phased out the misnomer Hurthle/Hürthle cell [4]. This revolutionary change, together with implementation of neuroendocrine IHC and standardization of the diagnostic chain proposed here (see Figure 2), seems to be a basic condition for further progress: “*Common language—all that is needed for successful work*”. 

This was the reason for the drastic reduction in the number of studies included in our review (Figure 1), and the data obtained (Table 1, Table 2 and Table 3) in fact came predominantly from comprehensive case reports. However, searching for pathogenetic connections with cause-and-effect relationships over statistical correlations is a completely different methodology [22,125] (Table 4). Case–control studies and time-lapse analysis in case reports resemble the paradigms of experimental medicine, e.g., animal studies (Table 4, adopted from ref. [125]).

When the PRISMA method was used, most crucial and clinically important data, as well as case reports, were not included by Coca-Pelaz A et al. [22]. Of note, this systematic review and meta-analysis was based on only 27 papers, including data from 1206 patients, between 2000 and 2020. Despite the aim to investigate the main characteristics of HCC, the meta-analysis revealed only gender, pathological data (e.g., tumor size, node involvement, extension without TNM), and the mode of medical interventions. Of the 27 publications, only a few contained information about survival without the clinical characteristics of HCC [22]. Interestingly, pooled results for overall (OS) and disease-free survival (DFS) at 10 years revealed lower OS than DFS (81% and 91%, respectively), with only OS showing a significant decrease (by 10%). 

This is a further argument in favor of a standardized framework approach to diagnostics, including the immunoendocrine background (paraneoplastic syndromes) (Figure 2), since as many as 10% of patients died between 5 and 10 years without tumor-specific symptoms (“disease-free” patients). This finding was confirmed by a unique observation by Kushchayeva Y et al., who reported a cancer-specific mortality (CSM) in HCC after 10 years of 51% [126]. Therefore, the high aggressiveness of HCC (misidentified as compromised survival) is a flawed paradigm [124] since (1) cause of death, CSM, and TNM are rarely published, (2) OS was determined by contact with families or data from registry [9,101,124,127]), and (3) patients were not observed in time-lapse sequences, e.g., with various follow-up appointments (i.e., between 10 and 160.8 months in the meta-analysis by Coca-Pelaz et al.) [22]. These findings indicated the need for an autopsy (Figure 2A) and strict differentiation (Figure 2B) in HCC.

Artificial intelligence (AI) models learn from a vast corpus of thyroid cancer (or DTC) texts and literature, which are full of discussions about limited data and “management.” AI can describe tactical recommendations in the language of a TC oncology and statistics, not the language of medicine (cause–effect relationship). If the model is trained on thousands of pages of this style of reasoning, it can reproduce these biases without critical and comprehensive insight, stalling the advancement of scientific knowledge and propagating misrecommendations (see graphical abstract).

## 7. Conclusions

In the AI era, fundamental biases in data collection, terminology (OA/OC vs. HCC/HCA, DTC), and linguistic areas (e.g., Hürthle or Hurthle vs. Hurtle) can cause unpredictable side effects. The key to formulating guidelines depends on literature selection. Due to myths and biases, in our opinion, establishing strict and adequate recommendations for HCC is currently impossible unless a reproducible diagnostic chain (presented in Figure 2) is implemented that prioritizes standardization and precision medicine without bias [30,122]. For example, the Polish Cancer Registry’s comparison of 20th century (1986–2000) and current data (2010–2024) showed that TC prevalence was previously significantly lower (half the size), but the percentage was similar [101]. This was despite the Chernobyl nuclear power plant disaster, which took place on 26 April 1986 and was the worst nuclear accident in history, releasing approximately 400 times more radiation (especially ^131^I) than the bombing of Hiroshima. Its effects were felt not only by residents of nearby areas but also by people in Poland. The two-fold difference in TC incidence was, therefore, a result of current fast diagnosis (like other cancers) and low availability of diagnostics before Poland’s accession to the European Union.

The spectrum of TC diagnostics, from the beginning (e.g., TFTs, imaging) to the end (cause of death in autopsy), requires standardization, differentiation, and holistic medicine. In HCC, the set of basic laboratory tests proposed herein (Figure 2) may reveal the unique features of HCC (e.g., hypercalcemia, IGT) (Table 1). Similarly, we suggest more frequent performance of PET-CT as an alternative of overused FNAB (e.g., staging and evaluating lymph node involvement, “reverse flip-flop” phenomenon) (Table 2).

To date, oncocytic cell thyroid cancer (or Hürthle cell carcinoma) has been treated according to generalized standards (e.g., surgery, TSH-ST, RAI), but this review underscores the need for precision medicine using new approaches and clinical methodologies, such as the observation of neuroendocrine and paraneoplastic presentations. These manifestations usually precede the development of cancer and are some of the determinants of OS and QoL. This approach is valuable given that traditional histology was not associated with OS in a multivariable analysis [127].

It is important to consider and correlate information across the patient’s clinical history, laboratory metrics, and various imaging (especially scintigraphy), and strict (molecularly or immunochemistry confirmed) histopathological analysis.

## Figures and Tables

**Table 2:** Various types of medical imaging used in oncocytic thyroid tumors.

Technique	Pros	Cons	Reference
Ultrasonography (US)	—cost-effective —detection of non-palpable small tumors —non-(radio)-toxic —safe (pregnant women) —examination of lymph node and focus of potential other oncocytic tumors *	—only one malignancy parameter ** —time-consuming algorithm —ultrasound examinations in the asymptomatic general population	[39] [40] [7]
FDG-PET/CT imaging	—shows hypermetabolic activity and glucose uptake —low background —full-body scan —“reverse flip-flop”	—low value in HCC/HCA differentiation —uneconomical —radiation exposure	[45] [49] [7]
Scintigraphy (imaging with 200 mCI ^131^I)	—more specific for TC than PET —differentiation of TC and thyroid metastases (e.g., from oncocytic parathyroid cancer) * —full-body scan (mediastinum metastasis was observed)	—low radioiodine avidity of HCC (common cold nodules) —radiation exposure —abolishes thyroxin substitution	[45] [54] [55] [46] [20]
Scintigraphy (technetium-99-based)	—99mTc selectively concentrated in the stomach, thyroid, and salivary glands —various radiopharmaceuticals —postoperative remnant thyroid tissue visualization	—radiation exposure —common cold nodules —low differentiation between HCC/HCA —parathyroid cancer	[32] [56] [55] [46] [47]

The table shows the possibilities of using various techniques for differentiating Hürthle cell cancer and presents the results of research conducted so far in this field, pointing to the advantages and disadvantages of individual solutions. * Oncocytic tumors arising from salivary glands (i.e., Warthin’s tumor), adrenal cortex, and parathyroid glands. ** The only degree of hypoechogenicity is sign of malignancy. It has been proven primarily for PTC. Abbreviations: FDG-PET/CT—^18^F-fluorodeoxyglucose positron emission tomography with computed tomography, TC—thyroid cancer, HCA/HCC—Hürthle cell adenoma/carcinoma, PTC—papillary thyroid cancer.

**Table 3:** Fine-needle aspiration biopsy (FNAB) use in thyroid nodules and Hürthle cell carcinoma: (pros) and (cons).

	Pros	Cons	Ref
Safety of the procedure	small area of damage (low risk)	few data (low benefit)	
Aim	nodular disease screening and shorten procedure duration	diagnostic purpose only	
Technique	easy technique: can be performed in any conditions	difficult assessment, frequent misinterpretation, especially in the case of atypical tumors	[54] [67] [72]
Results	cost-effective, quick result	inconclusive, especially with low cellularity, misdiagnosis (recommended core biopsy)	[76] [54]
Consequence	making decision about surgery easier (overuse)	postponing surgery and final diagnosis for benign disease, spread of HCC may be facilitated	[77] [78]
Malignancy (HCC vs. HCA)	molecular tests	non-discriminative in mild/malignant oncocytic cancer * low rate of pre-operative diagnosis of well-differentiated thyroid carcinomas	[60] [78] [79] [67] [80] [77]

* Observation of angio-/capsular invasion impossible. FNAB should be preceded by a careful analysis, not by following algorithms. Carefully weighing the pros and cons is very difficult in Hürthle cell cancer. Each doctor should decide whether they suspect a benign lesion (nodule) or an aggressive one. There are many benign thyroid lesions associated with HC or HC changes. It may be difficult for clinicians to discern the significance of these findings and to determine an appropriate course of action [80]. Abbreviations: Fine-needle aspiration biopsy (FNAB); Hürthle cell adenoma/carcinoma (HCA/HCC).

**Table 4:** Various (doubtful) interpretations and data collection (misselection) from a previous cohort [22] and comprehensive case study [15] in HCC using non-standardized methodology and nomenclature (i.e., incompatible with the 5th edition of the WHO Classification of Endocrine Neoplasms).

	Cohort/Meta-Analysis (e.g., [22])	Case–Control/Case Report (e.g., [15])
Study of rare diseases or rare constellations (here HCA/HCC) *	Impractical	Useful
Application of the clinical strategy	Indirect (current diagnostics and treatment of HCC based on existing standards for other thyroid cancers)	Direct (learning through imitation)
Nature of the study	Prospective or retrospective (metanalysis) *	Retrospective
Comprehensive description of natural history of the disease	No (usually demographic data, OS/DFS, gender)	Yes ** (e.g., cause of death)
Timeline of therapeutic regimens	No (e.g., HCC surgery, TSH-ST, RAI in separate studies)	Yes (collectively described)
Analyzed factors	Limited (due to lack of standardization) * Some of the reported parameters not available in all studies (e.g., TT3/4, TSH, TNM).	multiple (holistic) (i.e., glycemia, IGT, Ca^2+^)
Cause–effect relationship (association between exposure and an event)	Statistical correlation (probability)	Temporal, pathogenetic links (e.g., TFT evolution)
Patients	NON-homologous big group (frequent cancer e.g., FTC)	Homologous (comparable situation)
Results comparable with experimental study **	No (results are statistical risk only)	Yes (direct) **

The two different data collections and interpretation, as well as the approach to the patient with HCC, are exemplified in three publications. * In the case of HCC and rare diseases, retrospective observations (non-standardized data collection) dominate, as prospective observations require tedious, long-term, and multi-center studies. It is not surprising that only one parameter (TG level) was considered as a symptom of HCC. ** Thinking about translational medicine and the connection between experimental and clinical data, cohort tests based on statistical differences (e.g., between drugs or placebo) are not a good bridge. Transposition of the scientific knowledge is very difficult. However, a case study with a significant event (e.g., radiation exposure, surgery) or laboratory test abnormality (e.g., TSH, TT3/4 level) with a plausible time relationship (TC) resembles an experiment (e.g., Charnobyl disaster—see Section 7). Transferring all data (i.e., therapeutic regimen, TFTs, see Table 1) and direct comparison with animal results is feasible provided that it is a comprehensive publication [15], unlike ref. [31], which provided demographic or anthropometric data only (see Section Data Analysis (Bias Elimination): An Attempt to Collect and Unify Good Clinical Practices). Abbreviations: disease-free (DFS) and overall survival (OS), follicular thyroid cancer (FTC); Hürthle cell adenoma/carcinoma (HCA/HCC); impaired glucose toleration (IGT); thyroid function tests (TFTs); TNM classification of malignant tumors (TNM); thyroid-stimulating hormone suppression therapy (TSH-ST), thyroid cancer (TC).

## Data Availability

No new data were created or analyzed in this study.

## Abbreviations

The following abbreviations are used in this manuscript:

FDG-PET/CT or PET-CT	^18^F-fluorodeoxyglucose positron emission tomography with computed tomography
BHD	Birt–Hogg–Dubé
BMI	Body mass index
CT	Calcitonin
CSM	Cancer-specific mortality
DFS	Disease-free survival
DDT	Disease-directed therapy
FNAB	Fine-needle aspiration biopsy
FT4/3	Free tetra-/tri-iodothyronine
HCN	Hürthle cell neoplasm
IGT	Impaired glucose tolerance
IGF-1	Insulin-like growth factor 1
MEN	Multiple endocrine neoplasia type
NSE	Neuron-specific enolase
NE	Neuroendocrine
OA/HCA	Oncocytic/Hürthle cell adenoma
OC/HCC	Oncocytic/Hürthle cell carcinoma
OGTT	Oral glucose tolerance test
OS	Overall survival
PCC	Patient-centered care
PFS	Progression-free survival
QoL	Qualify of life
RIA	Radioiodine-131 ablation
RXR	Retinoid X receptor
TG	Thyroglobulin
TC	Thyroid cancer
FTC/PTC/DTC	Follicular/papillary/differentiated thyroid cancer
TTF-2	Thyroid transcription factor
TSH	Thyroid-stimulating hormone
TRE	Thyroid-hormone response element
TIRADS	Thyroid Imaging Reporting and Data System
TSH-ST	Thyroid-stimulating hormone suppression therapy
T4	Thyroxin
TT4/3	Total tetra-/tri-iodothyronine
TT	Total thyroidectomy
TNM	TNM classification of malignant tumors
US	Ultrasonography
WBS	Whole-body ^131^iodine scintigraphy
